# Strategies for Electrochemically Sustainable H_2_ Production in Acid

**DOI:** 10.1002/advs.202104916

**Published:** 2022-01-12

**Authors:** Yuxi Hou, Jiangquan Lv, Weiwei Quan, Yingbin Lin, Zhensheng Hong, Yiyin Huang

**Affiliations:** ^1^ Fujian Provincial Key Laboratory of Quantum Manipulation and New Energy Materials, College of Physics and Energy Fujian Normal University Fuzhou 350117 China; ^2^ Fujian Provincial Engineering Technology Research Center of Solar Energy Conversion and Energy Storage Fuzhou 350117 China; ^3^ Fujian Provincial Collaborative Innovation Center for Advanced High‐Field Superconducting Materials and Engineering Fuzhou 350117 China; ^4^ College of Electronics and Information Science & Organic Optoelectronics Engineering Research Center of Fujian's Universities Fujian Jiangxia University Fuzhou Fujian 350108 P. R. China

**Keywords:** acid, electrochemical, H_2_ production, sustainability, water electrolysis

## Abstract

Acidified water electrolysis with fast kinetics is widely regarded as a promising option for producing H_2_. The main challenge of this technique is the difficulty in realizing sustainable H_2_ production (SHP) because of the poor stability of most electrode catalysts, especially on the anode side, under strongly acidic and highly polarized electrochemical environments, which leads to surface corrosion and performance degradation. Research efforts focused on tuning the atomic/nano structures of catalysts have been made to address this stability issue, with only limited effectiveness because of inevitable catalyst degradation. A systems approach considering reaction types and system configurations/operations may provide innovative viewpoints and strategies for SHP, although these aspects have been overlooked thus far. This review provides an overview of acidified water electrolysis for systematic investigations of these aspects to achieve SHP. First, the fundamental principles of SHP are discussed. Then, recent advances on design of stable electrode materials are examined, and several new strategies for SHP are proposed, including fabrication of symmetrical heterogeneous electrolysis system and fluid homogeneous electrolysis system, as well as decoupling/hybrid‐governed sustainability. Finally, remaining challenges and corresponding opportunities are outlined to stimulate endeavors toward the development of advanced acidified water electrolysis techniques for SHP.

## Graphical abstract

This work provides the fundamental principles and summarizes several innovative strategies to access sustainable H_2_ production from acidic electrochemical water electrolysis.

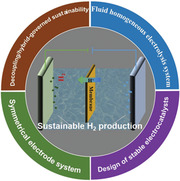

## 1 Introduction

Replacement of traditional fossil fuels with various clean renewable energy sources has become increasingly necessary and pressing in the face of current energy crises and environmental issues.^[^ ^1^, ^2^ ^]^ Renewable energy resources, such as wind, solar, and geothermal energy, have thus been widely explored in the past decades.^[^ ^3^ ^]^ However, they are discontinuous and intermittent, which largely limit their stable electricity output. Electricity storage systems that convert electrical energy into chemical energy, such as chemical carriers (e.g., H_2_,^[^ ^4^ ^]^ alcohol,^[^ ^5^ ^]^ and NH_3_^[^ ^6^ ^]^) and batteries (e.g., Li‐ion battery^[^ ^7^ ^]^), have been developed. Among these carriers, hydrogen has a high gravimetric energy density of 142 MJ kg^–1[^ ^8^ ^]^ and thus can be used in diversified and large‐scale applications, e.g., combustion devices,^[^ ^9^ ^]^ fuel cells,^[^ ^10^ ^]^ and the synthesis of industrial raw materials.^[^ ^11^ ^]^ Importantly, it can be used for energy generation and utilization without the emission of any greenhouse gases.^[^ ^12^, ^13^ ^]^ Electrochemical water electrolysis for H_2_ production has thus attracted extensive research interests. The process normally involves two half reactions: hydrogen evolution reaction (HER) at the cathode and oxygen evolution reaction (OER) at the anode. These two reactions can be accelerated in either acidic or alkaline media using a variety of catalysts. Water electrolysis is significantly more advantageous in acidic than in alkaline media because of the higher ionic conductivity, faster H reaction dynamics, and absence of CO_2_ contaminant, which leads to higher current densities and higher‐purity H_2_.^[^ ^14^, ^15^ ^]^ Typically, current densities are higher by a factor of 4–5 in electrolyzers operating in acidic than in alkaline media.^[^ ^14^ ^]^ More importantly, acidic electrolyzers have higher stability against shutdowns and load‐cycling.^[^ ^2^ ^]^ Therefore, water electrolysis in acidic media is a more promising approach for H_2_ production.

The deployment of water electrolysis in acidic media is hindered by a critical problem: the severe degradation of catalysts and electrodes in acidic media, especially at the anode where OER occurs and a strongly oxidative environment is formed.^[^ ^14^ ^]^ Most advanced alkaline OER electrocatalysts reported thus far, including transition metals and nonmetal materials, corrode and degrade to different degrees in oxidative environments with strongly acidic electrolytes.^[^ ^16^ ^]^ According to Sabatier's principle, Pt‐based and Ir/Ru‐based electrocatalysts are known to facilitate, with fair stability and excellent activity, acidic HER and OER, respectively.^[^ ^12^, ^14^, ^15^ ^]^ However, these noble metal electrocatalysts are not entirely stable in acidic media. Specifically, IrO_2_ suffers from slow dissolution in acidic media, and RuO_2_ is even less stable than IrO_2_.^[^ ^2^ ^]^ Hence, continuous and stable H_2_ production without substantial reduction, known as sustainable H_2_ production (SHP), from water electrolysis in acidic media is largely limited by the instability of electrode catalysts. Recently, Ir‐ and Ru‐based perovskite oxides with improved stability have been developed,^[^ ^17^ ^]^ and the tunable electronic structure of Ir and Ru was found to favor acidic OER. However, certain components of perovskite oxides (e.g., alkaline earth metal elements) are prone to leaching and the degraded oxides reconstruct and/or amorphorize under acidic OER conditions, causing performance degradation.^[^ ^18^, ^19^ ^]^ To achieve SHP, current main‐stream studies are focused on tuning the atomic/nano structure of metal electrocatalysts for enhanced stability and activity under acidic OER conditions, with only limited effectiveness.^[^ ^14^, ^15^ ^]^ Additionally, new reaction control strategies have been investigated (e.g., decoupling/hybridizing/tandem catalyzing strategies),^[^ ^1^, ^12^, ^20^, ^21^ ^]^ although the corresponding guiding principles for SHP based on water electrolysis in acidic media have been not summarized. Furthermore, a systems approach that considers operating reactions and electrode and device configurations/operations is still lacking, which is of great significance because it may provide innovative strategies for SHP. To implement this systems approach, a comprehensive understanding of SHP is indispensable.

In this review, we summarize the fundamental principles of SHP based on water electrolysis in acidic media. Then, we discuss recent advances and developments of associated catalysts, electrodes, and devices to propose several innovative strategies for SHP. These strategies entail the design of stable electrocatalysts, symmetrical electrodes, homogeneous fluid electrolyzers, and decoupling/hybrid controls. Finally, we outline the current challenges and future opportunities of these strategies and provide an outlook on advanced water electrolysis systems for SHP.

## 2 Fundamental Principles for SHP

**Figure 1:**
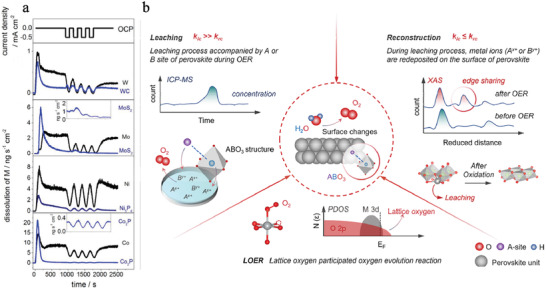
a) Metal dissolution profile in relation to applied potential for Co/Co_2_P, Ni/Ni_5_P_4_, Mo/MoS_2_, and WC/W. Reproduced with permission.^[^ ^29^ ^]^ Copyright 2017, Wiley‐VCH. b) Surface changes of ABO_3_‐type perovskite during OER and corresponding characterization. Red, blue, and purple spheres signify the oxygen, B, and A sites, respectively. Reproduced with permission.^[^ ^32^ ^]^ Copyright 2021, American Chemical Society.

SHP first requires highly active and stable catalysts, which depends on their surface active sites. Therefore, an in‐depth understanding of the evolution of surface active sites is essential. In a typical acidified water electrolysis reaction, the reactants, H^+^/H_2_O, are adsorbed onto the active centers and various intermediates (e.g., *H, *OH, *O, and *OOH) are generated via successive proton and/or electron transfer steps^[^ ^22^, ^23^ ^]^ to form the products, H_2_ and O_2_. The active sites on these catalysts are usually unsaturated sites, charge‐unbalanced sites, and vacant d‐orbital sites.^[^ ^23^, ^24^, ^25^, ^26^ ^]^ During water electrolysis, these active sites adsorb the intermediates and interact with them to varying degrees to facilitate proton‐electron transfer reactions,^[^ ^24^, ^27^ ^]^ which makes them vulnerable to further interactions.^[^ ^25^ ^]^ As a result, various surface reconstructions, such as phase transformation, composition leaching, metastable‐intermediate formation, and atom rearrangement occurs under the reaction conditions.^[^ ^25^ ^]^ In particular, acidified water electrolysis in low pH environments and low/high potentials aggravates these surface structural/chemical processes.^[^ ^26^, ^28^, ^29^, ^30^ ^]^ It is suggested that most electrocatalysts, including metals, nonmetals, and most noble metals, partially oxidize into their oxide/ion forms during OER, accompanied with possible dissolution in acid media.^[^ ^31^ ^]^ Likewise, they partially reduce into their low chemical valence state forms during HER.^[^ ^25^ ^]^ As illustrated in **Figure** **1**a,b, highly acidic electrolytes cause oxidative corrosion and dissolution of components, thereby inducing the loss of surface active centers and surface reconstruction during water electrolysis even for some robust compounds.^[^ ^32^ ^]^ In general, complex interactions between active centers and intermediates/electrolytes under strong electric fields lead to surface reconstruction and thus activity decay. More importantly, such interactions exist in many cases, making the phenomena inevitable. Understanding of this situation enables researchers to make multi‐faceted efforts for SHP, including catalyst design.

Vast research efforts have been devoted to retarding or accelerating surface reconstruction to obtain highly stable electrocatalysts for SHP.^[^ ^27^, ^30^, ^33^ ^]^ We have previously summarized these approaches, which include protective‐layer isolation, interface coupling, defective/atomic modulation, and pre‐synthesis of optimized structures.^[^ ^25^ ^]^ Some surface local structures (e.g., defects and vacancies) act as intrinsically unstable factors for surface reconstruction because the electronic structures of adjacent active centers change during water electrolysis.^[^ ^34^ ^]^ Thus, effective material designs should aim to protect the active centers of electrocatalysts and also to avoid the formation of these labile local structures. Understanding the reaction mechanism of HER and OER further facilitates the control of surface reconstruction and design of catalysts. The HER can proceed via either the Volmer‐Tafel (H^+^+e^−^+*→H*→H_2_) or the Volmer‐Heyrovsky pathway (H^+^+e^−^+*→H*; H^+^ +e^−^+H*→H_2_),^[^ ^35^ ^]^ and its reaction kinetics is faster than that of OER. More extensive surface reconstruction and activity decay usually occur at the anode because of the sluggish OER kinetics;^[^ ^36^, ^37^ ^]^ thus, more attention should be paid to this side. OER can proceed via the adsorbate evolution mechanism (AEM), the coupling of two metal–oxyl radicals (I2M), or the lattice oxygen evolution mechanism (LOEM).^[^ ^26^, ^38^ ^]^ In the AEM, H_2_O molecules, the reactants, dissociate into intermediate *OH and then *O, which undergoes nucleophilic attack by another H_2_O molecule to generate intermediate *OOH, ultimately leading to O_2_ formation and desorption. Most catalysts can facilitate OER via AEM. In I2M, the OER using catalysts proceed via O–O bond formation involving the combination of two metal–oxyl species to generate a peroxyl species and then O_2_. Binuclear metal catalysts, such as binuclear Ru or Mn catalysts, can catalyze the OER via the 12M approach.^[^ ^39^ ^]^ Scaling relations exist in these OER steps, which makes it difficult to optimize the adsorption of these intermediates simultaneously, leading to an inefficient OER.^[^ ^27^ ^]^ As for the LOEM, the lattice oxygen in the electrocatalysts can directly participate in the OER, with the formation of possible intermediates *OO and *OH.^[^ ^26^ ^]^ It is suggested that the O‐vacancy content significantly affects the OER via LOEM.^[^ ^40^ ^]^ Thus, O vacancies should be carefully considered when balancing activity and stability. Either way, a bias higher than 1.23 eV is usually needed to overcome these thermodynamic hindrance and to initiate the OER, leading to an electron deficient state of the electrode/electrocatalyst and other types of surface reconstruction, e.g., components leaching and phase transformation even for highly stable perovskite catalysts.^[^ ^32^ ^]^

**Figure 2:**
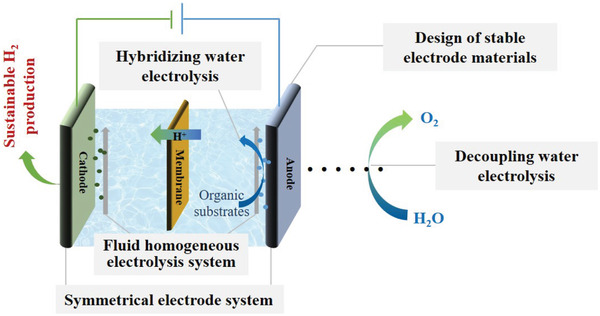
Strategies for SHP based on acidified water electrolysis.

In addition to the main unstable factor originating in the electrode material, the reaction and device configuration/operation are also of great importance. Therefore, systematic investigations of these aspects should also be conducted for SHP, as shown in the **Figure** **2**. First, the stability of acidified water electrolysis is strongly reliant on the electrode materials; thus, multi‐scale designs, e.g., morphology/phase controls and optimizing the electronic/atomic/nano structures of electrode materials with minimization of Gibbs free energy change in the corrosion/dissolution reaction,^[^ ^41^ ^]^ is still important for achieving SHP. Second, a large amount of active noble metals is usually needed for OER, whereas a small quantity of noble metals, such as Pt, leads to efficient HER activity. Because acidified water electrolysis under long‐term operation suffers from anode‐metal dissolution,^[^ ^25^ ^]^ followed by electro‐migration and electrodeposition on the cathode, it is thus possible to recover OER activity while retaining HER activity by exchanging the anode and cathode in a symmetric electrode system. Third, the dissolution of heterogeneous catalysts can be prevented by using a homogeneous electrocatalyst that is easily adsorbed on the electrode substrate. In addition, a continuous supply of homogeneous electrocatalysts can be facilitated by designing new flow electrolysis cells. Finally, the electrode reaction can be adjusted to lower the anode potential. For example, electro‐oxidation of some organic substrates, such as 5‐hydroxymethylfurfural,^[^ ^42^ ^]^ has favorable thermodynamics, and thus the anode potential is lower relative to OER. In hybrid water electrolysis systems, anode corrosion can be mitigated for the efficient production of H_2_ and value‐added organic products.^[^ ^1^ ^]^ Similarly, the decoupled water electrolysis strategy can be applied to mediate HER and OER,^[^ ^12^ ^]^ and better stability can be achieved by independently optimizing the decoupling agents and catalysts. In what follows, we discuss the recent advances in these four strategies and resolve their related features, merits/demerits, and current situation.

## 3 Design of Stable Electrocatalysts

Designing stable electrocatalysts is key to accessing SHP based on acidified water electrolysis. In many cases, however, there is a trade‐off between activity and stability.^[^ ^43^ ^]^ In particular, an inverse relation between activity and stability has been observed for some well‐established electro‐catalysts (e.g., IrO_2_ is less active but more stable than RuO_2_) during the OER in acidic media.^[^ ^43^ ^]^ For design of robust electrode materials, various approaches, such as support stabilization, forming protection layers, morphology and phase controls, and modulation of electronic/atomic/nano structures, can be employed. In the following part, we will discuss the advances of these approaches in relation to promoted stability.

### 3.1 Electrocatalysts for Acidic HER

**Figure 3:**
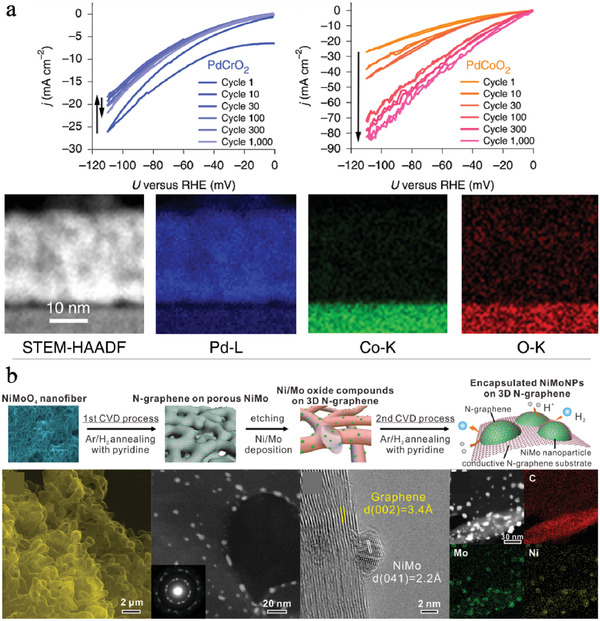
a) Cathodic cycling of PdCrO_2_ and PdCoO_2_ in sulfuric acid solution and high‐angle annular dark field scanning transmission electron microscopy (HAAD‐ STEM) image and energy dispersive X‐ray spectroscopy (EDS) of post‐catalysis PdCoO_2_. Adapted with permission.^[^ ^53^ ^]^ Copyright 2019, Nature Publishing Group. b) Preparation processes and characterization of NiMo nanoparticles coated with N‐doped graphene. Adapted with permission.^[^ ^56^ ^]^ Copyright 2018, American Chemical Society.

Although Pt is regarded as the benchmark electrocatalyst for HER in acidic media, the high cost of Pt has activated investigations into low‐cost noble or non‐noble metals. One of the simplest approaches to access low‐cost noble metals with reservation of acidic HER activity is preparation of single atom Pt, of which the average oxidation state has strong correlation with the Pt–H interaction, thus tuning the HER activity.^[^ ^44^ ^]^ The optimal oxidation state of Pt was evaluated to be ≈+2. By a self‐reduction method to approach this oxidation state, we can obtain a high HER activity of the single atom Pt catalyst with overpotential of only 25 mV at 10 mA cm^–2^.^[^ ^45^ ^]^ The preparation of stable single atom Pt requires strong metal‐support interaction, by which the high‐energy barrier would limit Pt diffusion on the surface to form clusters/nanoparticles, thus demonstrating the structural stability.^[^ ^44^ ^]^ Among the other noble metals reported thus far, metallic Ru loaded on triazine‐ring‐doped carbon, multi‐walled carbon nanotubes, or edge‐carboxylic‐acid‐functionalized graphene nanoplatelets have shown great potential for HER in acidic media.^[^ ^46^, ^47^, ^48^ ^]^ However, the supported Ru‐based catalysts suffer from particle aggregations arising from the migration and deposition of Ru‐based particles partly because of insufficient interactions with the support.^[^ ^49^ ^]^ To overcome this problem, Li et al. employed a polydopamine‐reduced process to apply a Ru coat by exploiting the interaction between –NH_2_ and Ru.^[^ ^50^ ^]^ The coated PDA was further converted into an N‐doped carbon (NC) layer after annealing. Consequently, the obtained Ru@NC catalyst showed not only high HER activity in acidic media with an overpotential of only 27.5 mV (10 mA cm^–2^) but also excellent long‐term stability from the protective effect of the NC shell. Similar to Pt atomization, single atom Ru could be obtained by its anchoring on CoP nanoparticles, with excellent stability as demonstrated by the negligible current decay during a 20 h constant potential test.^[^ ^51^ ^]^ Pd‐based compounds are well‐researched noble‐metal HER catalysts.^[^ ^52^ ^]^ In multicomponent Pd‐based compounds, the component with the lower electronegativity may suffer from leaching and dissolution during HER in acidic media, which is also affected by the chemical and electronic structure of the compounds. For example, the PdCrO_2_ surface is more stable than the PdCoO_2_ surface during the potential cycling test (see **Figure** **3**a), with the latter leaching Co from the lattice.^[^ ^53^ ^]^ Therefore, such surface reconstruction needs to be further correlated with HER activity. In the above case, the cycling experiment causes surface reconstruction of PdCoO_2_, thus forming a strained Pd coating layer. The intrinsic electrocatalytic mechanism is altered by this strained Pd coating layer to induce the formation of highly active *β*‐palladium hydride for efficient HER. In addition to Ru and Pd metal materials, electrocatalysts based on Rh^[^ ^54^ ^]^ and Ag^[^ ^55^ ^]^ have been synthesized and optimized with activity and stability on par with commercial Pt/C.

Various non‐noble‐metal materials based on Mo and containing carbide, phosphide, sulfide, nitride, or selenide, have been widely studied for HER in acidic media. Although they are more stable (corrosion resistant) in acidic than in alkaline media,^[^ ^57^, ^58^, ^59^, ^60^ ^]^ their stability is still low relative to materials based on noble metals. For example, the active species of Mo sulfide dissolve and corrode in 0.5 m H_2_SO_4_.^[^ ^61^ ^]^ Therefore, several methods have been developed to further improve the stability of Mo‐based materials: 1) Dispersing Mo‐based materials onto substrates, such as carbon scaffolds, to enhance structural stability and electrical conductivity because of the strong interactions between catalyst and support.^[^ ^61^, ^62^ ^]^ Without strong interactions, however, the Mo compound can still peel off, e.g., Mo_2_C nanoparticles can shed from carbon fiber paper.^[^ ^57^ ^]^ 2) Fabricating unique architectures, such as nanowires, nanosheets, and nanocubes, to promote stability by mitigating volume expansion during HER in acidic media. Typically, Mo/Mo_2_C heteronanosheets with a large lateral dimension can efficiently maintain the active sites and electron transport channels at the heterointerfaces and prevent interface collapse and aggregation.^[^ ^59^ ^]^ 3) Intrinsically stable crystal structures are more preferred. For example, among the various Mo carbides (*β*‐Mo_2_C, *α*‐MoC_1−_ *_x_*, *γ*‐MoC, and *η*‐MoC), *β*‐Mo_2_C is regarded as the best catalyst for HER in acidic media in terms of activity and stability because its electronic structure is similar to that of noble metal Pt.^[^ ^58^ ^]^ For MoS_2_, the 1T phase is less stable and active than the 2H phase because of its strong binding with *H.^[^ ^63^ ^]^ 4) The electronic structure of Mo compounds can be modulated by incorporating heteroatoms, which significantly improves the corrosion resistance of active species. Typically, dopants (e.g., C, S, and N) that are more electronegative than P can stabilize the P^3–^ states of MoP nanoparticles via the formation of various nonmetal–nonmetal bonds.^[^ ^64^ ^]^ The electron density of P can be reduced by donating back to the vacant d‐orbital of Mo in the MoP nanoparticles. Consequently, stability and activity are promoted for HER in acidic media. Tungsten has the chemical properties similar to Mo, with even high stability in acidic media. It thus can serve as the stable substrate for active heteroatom modification. Recently, Han et al. prepared a single atom V loaded on 1T‐WS_2_ via the one‐step chemical vapor deposition method.^[^ ^65^ ^]^ The complex catalyst exhibited excellent stability for acidic HER due to the strong coupling of single atom V on the robust 1T WS_2_ structure.

Other transition metals, such as Ni, Co, and Fe‐based compounds except their soluble oxides, have also been studied for HER in acidic media.^[^ ^66^, ^67^, ^68^, ^69^ ^]^ Among them, Ni‐based nanomaterials have been extensively studied. For example, Ni_2_P/Fe_2_P arrays can be fabricated via a phosphating process to boost its HER activity in acidic media and also to suppress the significant Ni corrosion/dissolution in acidic media. However, individual Ni phosphide is unstable in H_2_SO_4_.^[^ ^70^ ^]^ Its corrosion tolerance can be promoted by increasing the P content in the materials without loss of HER activity.^[^ ^71^ ^]^ Typically, Ni_5_P_4_ is more resistant to corrosion in H_2_SO_4_ than Ni_2_P.^[^ ^71^ ^]^ A more stable form of Ni phosphide can be produced by coating it with carbonaceous layers. The aforesaid Ni_2_P/Fe_2_P arrays prepared from a Prussian blue analogue are protected with a carbon shell, which prevents the metal phosphide nanoparticles from aggregation and corrosion.^[^ ^70^ ^]^ Such protective carbon layers should be controlled to prevent activity loss. Ito et al. prepared a series of NiMo nanoparticles encapsulated by increasing numbers of N‐doped graphene layers for HER in acidic media, as shown in Figure 3b.^[^ ^56^ ^]^ It was found that acid resistance greatly improved but activity gradually reduced as the layer number increased. Post‐catalysis characterizations using electron microscopy, X‐Ray diffraction (XRD), and X‐ray photoelectron spectroscopy (XPS) revealed that after long‐term CV cycling, the N‐doped graphene layers were slightly broken, whereas the porous morphology and metal nanoparticles, including their original valence, were well preserved. This strategy of using a carbon layer as protection can be extended to Co‐based materials and to more diversified components for enhanced HER activity by modulating *H adsorption.^[^ ^72^ ^]^ During the pyrolysis preparation of Co‐based materials, multiple components involving single atom Co species were formed on carbons, and all components have beneficial effects for acidic HER.^[^ ^73^, ^74^, ^75^ ^]^ However, the complex active components make it difficult to explore the intrinsic properties of single atom Co species. Recently, there were two methods reported to obtain the pure single atom Co catalysts: one is the pyrolysis of Zn/Co‐containing metal‐organic framework compound;^[^ ^76^ ^]^ the other is laser irradiation to extract Co species and convert them to single atom Co.^[^ ^77^ ^]^ In both approaches, the single atom Co was firmly anchored by N coordination on the C lattice, forming the stable and active Co species. The heteroatom‐doped carbon materials (e.g., B, P, and S to N‐doped carbons) could not only be used as the supports for anchoring single atom metal, but also be served as active species for HER in acidic media,^[^ ^78^ ^]^ although their activity is lower than that of metal‐based materials, limiting their practical applications.

Overall, modifying noble metals (e.g., Ru and Pd) and non‐noble metals (e.g., Mo and Ni) produces catalysts with great stability for HER in acidic media. To reduce the application cost of noble metals, one can down‐size the particles and/or support them on carbon substrate. An efficient strategy for promoting the stability of all HER catalysts is to use a carbon layer as protection. With this protection layer, however, the active sites should be reevaluated, and the stability enhancement is strongly reliant on the properties of the carbon layer. The catalysts suffer from more severe dissolution once the carbon layer is broken.

### 3.2 Electrocatalysts for Acidic OER

**Figure 4:**
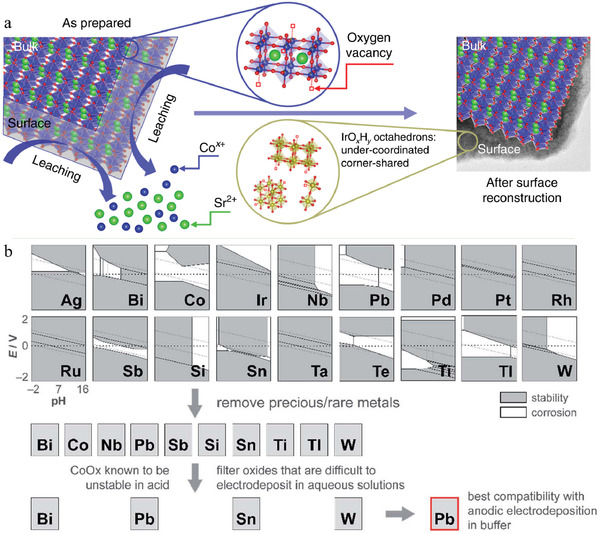
a) Schematic of surface reconstruction for SrCo_0.9_Ir_0.1_O_3−*δ*_. Reproduced with permission.^[^ ^85^ ^]^ Copyright 2019, Nature Publishing Group. b) Pourbaix diagram‐assisted analysis and screening of metal oxide, which is stable at low pH and high anodic potential. Reproduced with permission.^[^ ^92^ ^]^ Copyright 2020, Royal Society of Chemistry.

Most earth‐abundant materials cannot survive the strong acidic conditions of OER, except Ir‐ and Ru‐based materials.^[^ ^79^ ^]^ However, these noble metals are the scarcest elements in the Earth's crust, and Ir is tenfold scarcer than Pt.^[^ ^80^, ^81^ ^]^ Therefore, only a minimal amount of these elements should be used as electrocatalysts for OER in acidic media. A general and effective approach to enhance electrocatalytic activity/stability with minimal utilization is by forming alloys (e.g., IrNi or IrCo alloys^[^ ^80^ ^]^) or compounds (e.g., iridate compounds^[^ ^82^, ^83^ ^]^) with tunable metal–oxygen interactions via geometric and/or electronic modifications and by exposing the noble metal.^[^ ^84^ ^]^ One of the most studied systems is the Sr‐Ir complex oxides, Sr*_x_* Ir*_y_* O*_z_*, such as Sr_2_IrO_4_ and SrIrO_3_. In the oxide of Sr_2_IrO_4_, the Sr and Ir components suffer from dissolution during OER in acid media, and the dissolution rate slows down as a stable Ir‐rich surface forms after surface reconstruction.^[^ ^83^ ^]^ A similar phenomenon was also observed in the Sr‐Ir perovskite oxides, such as SrCo_0.9_Ir_0.1_O_3−*δ*_, as illustrated in **Figure** **4**a.^[^ ^85^ ^]^ Different perovskite phase structures have significantly different stabilities. Typically, 6H‐SrIrO_3_ surrounded by the [001] facet is more stable than the 3C‐phase SrIrO_3_ perovskite with a pseudo‐cubic structure; the latter undergoes surface amorphorization during OER in acidic media.^[^ ^86^ ^]^ A mechanistic study revealed that 6H‐SrIrO_3_ possessed the weaker Ir 5d–O 2p hybridization and weaker Ir–O covalent bond, thus preventing its structural loss and surface reconstruction during OER. Recently, Chung et al. systematically examined the structure–stability relation of these Ir‐based complex oxides.^[^ ^87^ ^]^ They found that the [IrO_6_] octahedra were connected to each other in the A*_x_* Ir*_y_* O*_z_* complex oxide with A in between, and they revealed the different connectivity between [IrO_6_] octahedra. Stability was also correlated with crystal structure, which was categorized into three types. Among these, the Ir oxides with face‐sharing connectivity and/or strong edge‐sharing showed the highest stability because the [IrO_6_]‐framework resisted collapse even with A‐cation leaching out over 600 anodic cycles.^[^ ^87^ ^]^ Heteroatom doping/modification can further promote the stability of Sr‐Ir and Ir oxides: 1) SrIrO_3_ doped with Ti is significantly more stable because metal leaching from the oxides during acidic OER is reduced;^[^ ^88^, ^89^ ^]^ 2) SrIrO_3_ doped with Zr (SrZrO_3_) weakens the metal–oxygen covalent bond, which suppresses surface metal dissolution (Sr and Ir) during OER in acidic media toward better stability.^[^ ^43^ ^]^ 3) Single atom Ag‐modified IrO*_x_* could enhance the Ir‐lattice O bonds, which combined with the lower overpotentials to reduce the loss of lattice O from LOEM and thus promoted the OER stability.^[^ ^90^ ^]^ Beyond the general approach discussed above, the other pathway to enhance activity/stability of Ir is atomization, especially with short‐range ordered Ir structure. Shan et al. have demonstrated that single atom Ir with short‐range order in the Ir_0.06_Co_2.94_O_4_ catalyst could downshift the d‐band center of Co species,^[^ ^91^ ^]^ suggesting the lower tendency of losing valence electron and being electrochemically oxidized toward better stability.

Ru is the cheapest of the Pt group metals, and therefore its application as an OER catalyst can be substantiated. However, pure‐phase Ru‐based catalysts, i.e., Ru or RuO_2_, can easily corrode and dissolve in acidic media;^[^ ^93^ ^]^ thus, they are less stable than IrO_2_ during OER in acidic media. Atomization of Ru is an effective strategy to promote stability, not only by allowing fine control over the electronic structure of Ru for better activity but also by providing additional binding effects to stabilize active Ru species. NC was used to bind Ru to form Ru–NC, which suffered only 5% atom loss during a 30‐h OER test.^[^ ^94^ ^]^ The slight shrinkage of the Ru–N bond used to fix the Ru atoms strongly to the carbon surface can prevent Ru dissolution during OER in acidic media. Defect sites can also be used to couple atomically dispersed Ru, as demonstrated by the unique periodic nano‐islands in Ru_1_–Pt_3_Cu,^[^ ^95^ ^]^ wherein aggregation and migration of Ru are prevented because of defect–Ru interactions. Furthermore, atomically dispersed Ru contributes less charge (≈0.19–0.21 e) than Pt_3_Cu moieties (≈0.54–0.59 e) to surface O species during OER. Thus, resistance against over‐oxidation and dissolution on Ru is enhanced toward better stability during OER in acidic media. Similarly, graphene, serving as an electron reservoir, can donate electrons to RuO_2_^[^ ^96^ ^]^ to prevent over‐oxidation and corrosion. Beyond atomic Ru, considerable research efforts have been devoted to Ru oxides in which stability is improved by morphology control and doping. Notably, several structurally robust RuO_2_ nanosheets have been prepared.^[^ ^97^, ^98^ ^]^ Nevertheless, component stability is poor due to partial dissolution in acid, further coarsening the Ru nanosheets. Thus, doping effects were used to achieve stability, and various elements have been incorporated into Ru oxides: 1) Incorporating yttrium into pyrochlore‐type Ru oxides can promote activity and stability by altering the energy levels of the Ru 4d orbital;^[^ ^99^, ^100^ ^]^ only 4% of Ru dissolved over 10 000 potential cycles in acid. Incorporating Zn into the Y_1.85_Zn_0.15_Ru_2_O_7−*δ*_ electrocatalyst also promotes activity without compromising stability.^[^ ^101^ ^]^ 2) A Cr_0.6_Ru_0.4_O_2_ solid solution formed by incorporating chromium has lower occupation at the Fermi level and lower Ru and Cr loss (< 2.5% and 8% after 10 000 potential cycles, respectively) without changes in crystallinity or morphology.^[^ ^102^ ^]^ 3) RuO_2_ co‐doped with Cu and Ca forms quadruple perovskite oxide CaCu_3_Ru_4_O_12_, which is a stable OER catalyst with only 2.9% Cu, 6.7% Ca, and 2.7% Ru loss during a 24 h continuous test.^[^ ^103^ ^]^ The Ru–O bond is weakened by element incorporation, which reduces lattice‐O participation during OER, thus enhancing catalyst stability during OER in acidic media. 4) Na‐doped ruthenium perovskite (Sr_1−_ *_x_* Na*_x_* RuO_3_) is more stable than SrRuO_3_ because the Ru>4+ and Sr ions are stabilized in the lattice, thus increasing its resistance to dissolution.^[^ ^93^ ^]^ 5) Single atom Ni‐doped RuO*_x_* was formed by hot‐acid leaching of Ni‐Ru@RuO*_x_*.^[^ ^104^ ^]^ During the leaching process, the unstable Ni species were removed, producing the abundant metal vacancies and thus rendering it better stability by limiting the proceeding of OER via LOEM. In general, component dissolution is related to catalyst stability. Although overall morphology and structure are maintained before and after testing, component dissolution still decreases catalyst activity during OER in acidic media.

Earth‐abundant non‐noble metals are cheaper than noble metals, although the former often suffers from relatively severe corrosion and dissolution during OER in acidic media. Therefore, finely controlling the pH to higher values (weaker acidic conditions) and the potential to lower values is necessary to prevent severe corrosion, as demonstrated by the corrosion diagram shown in Figure 4b.^[^ ^92^, ^105^ ^]^ For example, *γ*‐MnO_2_ can catalyze OER for over 8000 h without deactivation in the stable potential window between 1.6 and 1.75 V at the pH of 2.^[^ ^106^ ^]^ However, these controls decrease the efficiency of OER because conductivity and reactivity are lowered. Therefore, two strategies are required to improve the corrosion resistance of catalysts: enhancement of intrinsic stability and implementation of external stabilization effects. In the first strategy, typically, active Fe/Co/Ni/Mn metals are combined with Sb/Nb/Mo/W/Pb metals to form complex metal oxides with robust phase structures that are effective and stable during OER in acidic media, which is partially determined from the calculated Δ*G*_pbx_,^[^ ^92^, ^107^ ^]^ a parameter that indicates the phase–potential–pH relations and thus material stability. Among the various phase structures, many stable Mn‐based oxides exhibit the rutile structure. For example, Mn^+3^ is stabilized in a rutile‐type phase (Sb,Mn)O_2_,^[^ ^108^ ^]^ and the dissolution of active Ni/Mn is inhibited by forming the crystalline Ni*_x_* Mn_1−_ *_x_* Sb_1.6−1.8_O*_y_* rutile phase.^[^ ^109^ ^]^ Cohesive energy is another descriptor for stability; it represents the change in energy from free atoms without interactions to atoms in a solid. Recently, Kumta et al. incorporated Nb into the *α*‐MnO_2_ phase to increase the cohesive energy (more negative) because the Nb–O bond has a higher binding energy (726 kJ mol^−1^) than the Mn−O bond (362 kJ mol^−1^).^[^ ^110^ ^]^ Moreover, they further introduced F into the solid solution catalyst ((Mn*_x_* Nb*_y_*)O_2_:zF) to lower charge‐transfer resistance and to enhance electronic conductivity. Consequently, the stability and activity of (Mn_0.75_Nb_0.25_)O_1.375_F_0.625_ were promoted relative to *α*‐MnO_2_. The second strategy typically employs a hybrid complex by combining an active component with an acid‐stable structure. For example, active Fe oxides were incorporated into TiO*_x_* nanowires via Fe substitution and calcination.^[^ ^111^ ^]^ As a result, the current of this hybrid catalyst decreased only by 18.7% during an *i*–*t* test of OER in acid, whereas that of the Ti foam‐supported Fe_2_O_3_ decayed significantly, by 66.7%. Active atomic metal species facilitate such incorporations and also maximizes catalyst activity during OER. To stabilize the atomically dispersed metals (e.g., Co, Fe, and Ni), various carbons, such as O‐functionalized graphene nanosheets,^[^ ^112^ ^]^ hollow N‐doped carbon,^[^ ^113^ ^]^ N‐coordinated carbon nanofiber,^[^ ^114^ ^]^ and other heteroatom‐doped carbons,^[^ ^115^ ^]^ have been used. However, carbon oxidation and corrosion are unavoidable during OER in acidic media, although higher graphitization degrees and lower dopant contents can alleviate these issues. Beyond these stabilizers, cyano‐bridged coordination polymers,^[^ ^116^ ^]^ and polyoxometalates^[^ ^117^ ^]^ can be used. In particular, Galan‐Mascaros et al. prepared a new polyoxometalate electrocatalyst, [Co_9_(H_2_O)_6_(OH)_3_(HPO_4_)_2_(PW_9_O_34_)_3_]^16−^ (Co‐POM), for OER in acidic media.^[^ ^117^ ^]^ The active species in the Co‐POM cluster does not leach, and only a small amount of counter‐cations leaches out by ion exchange with H^+^, without affecting the overall catalyst activity during OER in acidic media.

In general, the stability of materials can be enhanced for OER in acidic media by controlling their elements, bonds, structure, and phase. The modification of noble metals (i.e., Ir and Ru), which are more resistant to corrosion than the non‐noble metals, is focused on stronger bonds and structures. The non‐noble metals are usually combined with new highly stable phases for OER in acidic media. In contrast, controlling the morphology of these OER catalysts has received less study. Promising morphologies, such as 1D architectures and 2D nanosheets, are considered during catalyst design because they can inhibit certain physical processes, e.g., avoiding the aggregation of catalyst arrays.^[^ ^118^ ^]^ Moreover, substrates with higher tolerance against acid corrosion at high potentials and with stronger adhesion with catalysts to prevent physical detachment in the presence of gas bubbles have been investigated. Enhancing material stability for both HER and OER with these designs can facilitate SHP.

## 4 Fabrication of Symmetrical Electrode System

**Figure 5:**
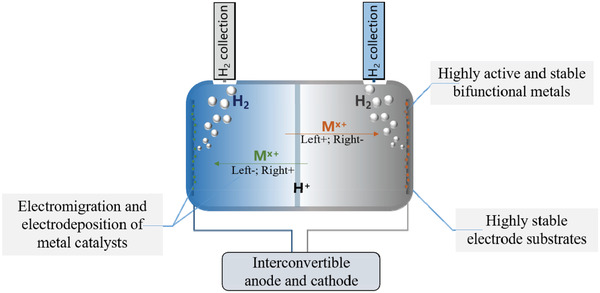
Schematic diagram for the fabrication of symmetrical electrode systems with interconvertible anodes and cathodes.

The above discussion shows that substantial improvements have been made to HER and OER catalysts operating in acidic media. However, stability issues, particularly of the anode, remains unsolved, thus limiting SHP to a large extent. Focusing on the development of catalysts is inadequate, and further systematic designs for water electrolysis in acidic media is indispensable. Considering that anode corrosion/dissolution and metal deposition on the cathode act together to degrade the overall performance of water electrolysis, one can utilize these processes and provide an in situ renewable anode during overall water electrolysis by exchanging the anode and cathode. For this design, a symmetrical electrode system with an interconvertible anode and cathode is required, as shown in **Figure** **5**. It requires the use of bi‐functional components based on the well‐developed HER/OER electrode materials. Bi‐functional electrode materials that have been developed comprise noble metals,^[^ ^119^ ^]^ non‐noble metals,^[^ ^120^, ^121^ ^]^ and even non‐metals,^[^ ^122^ ^]^ among which metal materials are the better candidates because of their higher activity/stability and the feasibility of in situ electrochemical synthesis. Beyond material selection, some fundamental principles should be considered in the design of interconvertible catalysts/electrodes: 1) The electrode substrate should comprise highly stable materials, among which graphite/Ti‐based compounds may be ideal choices. Electrode substrates provide stable surface sites for anchoring bi‐functional components during the electrode deposition reaction. 2) The synthesis of bi‐functional active materials should be adapted to the electrodeposition approach, which provides the basis for activity regeneration in the system by in‐situ electrodeposition. Thus, some materials, such as non‐noble carbons and metal‐non‐metal compounds, are not applicable. 3) The frequency of anode and cathode exchange should be as low as possible to collect high‐purity H_2_ from SHP. Highly stable bi‐functional active components are required after anchoring. 4) A high turnover frequency (TOF) of bi‐functional materials is necessary for fast SHP. In what follows, we discuss recent advances in catalyst candidates and the screening criteria of promising candidates, and we also propose strategies to improve the stability of symmetrical electrode systems.

**Figure 6:**
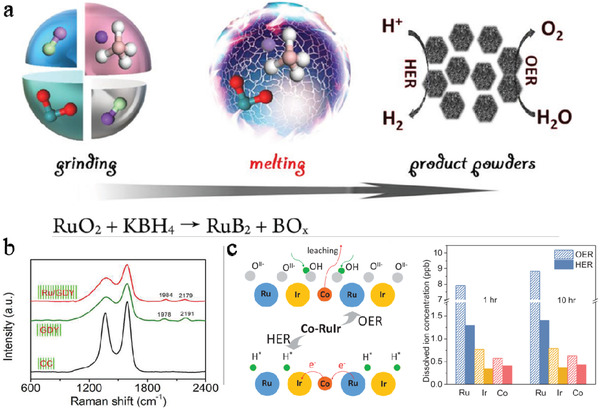
a) Illustration of the Synthesis and Structure of the RuB_2_ Catalyst. Adapted with permission.^[^ ^124^ ^]^ Copyright 2020, American Chemical Society. b) Raman spectra of Ru/Graphdiyne (GDY), GDY and carbon cloth (CC), respectively. Adapted with permission.^[^ ^128^ ^]^ Copyright 2020 Elsevier Ltd. c) Schematic of bifunctional mechanism for Co‐RuIr, and the ICP‐MS analysis for Co‐RuIr after stability measurements. Adapted with permission.^[^ ^129^ ^]^ Copyright 2019, Wiley‐VCH.

Among the various bi‐functional materials, Ru‐based materials have been widely studied because of their excellent OER catalysts and also their Pt‐like Ru–H bond for efficient HER. However, the component and structure of Ru‐based materials for the most active/stable OER and HER are different, hindering their applications in symmetrical electrode systems. For example, in acidic media, Ru has fair HER activity, whereas its OER activity is rather poor.^[^ ^123^ ^]^ RuO_2_ is regarded as a state‐of‐the‐art catalyst for OER, yet its activity for HER is unsatisfactory.^[^ ^123^ ^]^ More importantly, metallic Ru is not very stable in acidic media due to oxidation and dissolution during electrocatalysis.^[^ ^124^ ^]^ Moreover, RuO_2_ is unstable during OER, and O_2_ evolves via the LOEM.^[^ ^124^ ^]^ Therefore, these contradictory properties should be overcome before symmetrical electrode systems can be applied. Recently, some novel O‐free Ru‐based complexes have been developed. Mu et al. reported an ionothermal route using a salt melt of KCl and LiCl as the solvent to prepare RuB_2_ (see **Figure** **6**a).^[^ ^124^ ^]^ RuB_2_ is highly stable during water electrolysis because O_2_ evolution from lattice oxides is prevented, and it exhibits high activity with overpotentials of 52 and 223 mV for the HER and OER, respectively. Similarly, Ru‐Te materials were developed for water electrolysis in acidic media.^[^ ^123^, ^125^ ^]^ However, these Ru‐based metal‐non‐metal complex systems are not suitable as active materials of interconvertible anodes and cathodes because electrochemical co‐deposition of metal ions after dissolution is difficult. In this regard, metal–metal systems, e.g., Ru‐Ni complex with optimized d‐band interactions that weakens the binding of active sites with H and O for easier H–H and O–O formation,^[^ ^126^, ^127^ ^]^ exhibit more promise in such systems. During system operation, the deposited active metal species on the cathode usually presents as a single atom or atom clusters, and their desorption, corrosion, or ripening should be avoided. Thus, the strong coupling between substrate and active component combined with atomic structure optimization should be considered, as exemplified by the strongly coupled atomic Ru to C for efficient HER/OER via an electron‐mediating mechanism.^[^ ^128^ ^]^ The interaction could be revealed by various spectra characterizations, such as Raman spectra as seen in Figure 6b.

Iridium (Ir)‐based catalysts have OER and HER activities in acid.^[^ ^130^ ^]^ Similar to Ru, metallic Ir is more suitable for HER, whereas IrO_2_ is more active for OER.^[^ ^131^ ^]^ Recently developed advanced materials, such as Ir‐Se^[^ ^132^ ^]^ and Ir‐Te,^[^ ^133^ ^]^ have achieved integration of bi‐functional HER/OER activities, although these Ir‐based non‐metal catalysts are hard to prepare via in situ electrochemical synthesis. This bottleneck can be overcome by using Ir‐based catalysts. Among them, Ir‐Co materials^[^ ^134^, ^135^, ^136^ ^]^ have received more research interest. However, Ir‐Ni^[^ ^137^ ^]^ and Ir‐Al catalysts,^[^ ^138^ ^]^ show more potential for application in symmetrical electrode systems. The types of active metal components and the substrates are two key factors to be considered in designing interconvertible anodes and cathodes: 1) To achieve bi‐functional activity, a general approach is to integrate different active metals. For example, Integrating Pt and Co with Ir also produces an active catalyst, and a low voltage of only 1.53 V is required for overall water electrolysis in acid.^[^ ^139^ ^]^ Combining Co and Ru with Ir produces a highly active bifunctional catalyst, with overpotentials of only 14 mV for HER and 235 mV for OER at 10 mA cm^–2^.^[^ ^129^ ^]^ However, the element would suffer from slight dissolution especially during OER test (see Figure 6c). 2) Heteroatom‐doped graphitized carbons^[^ ^37^, ^140^ ^]^ and W‐based materials^[^ ^141^ ^]^ can be used as substrates because they are highly stable and their geometric and electronic structures are tunable, thus enabling further tailoring and optimization of intermediate binding.

In addition to Ru and Ir, Rh is another noble metal with bi‐functional activity for acidic HER/OER. The stability and bi‐functional activity of Rh can be further enhanced by combining it with non‐metal P^[^ ^142^ ^]^ or Cu,^[^ ^143^ ^]^ In particular, with the RhCu alloy in 0.5 m H_2_SO_4_ electrolyte, overpotentials of only 12 and 345 mV are required for HER and OER, respectively.^[^ ^143^ ^]^ These enhanced activities originate in the weakly adsorbed atomic O and H as well as the enhanced dissociation of H_2_O. However, Rh is very rare and more expensive than Pt and Ir. Therefore, it has no obvious cost advantage in large‐scale symmetrical electrode systems. Other non‐noble metal materials, such as N‐doped WC,^[^ ^144^ ^]^ Mo‐supported Co_9_S_8_,^[^ ^145^ ^]^ and single atom Co‐decorated MoS_2_,^[^ ^146^ ^]^ have been developed for overall water electrolysis in acid, although their bi‐functional activity and stability are still inferior to noble metals, and electrochemical methods cannot be used to prepare them, rendering them less feasible in symmetrical electrode systems.

In general, noble metal–non‐metal complexes, such as Ru–Ni and Ir–Co alloys, can serve as bi‐functional catalyst components for the symmetrical electrode systems. In particular, Ru‐/Ir‐based multicomponent alloys and even high‐entropy alloys (more than five components) may modulate electronic and atomic structures and are worth exploiting in future. There are two pathways to further enhance the stability of active metal components on the substrate: 1) Using organic modifiers that support coordination bonding with active metals can stabilize metal atoms/clusters. For example, cucurbit^[^ ^6^ ^]^uril (CB^[^ ^6^ ^]^) was used as a stabilizer to anchor Ir species by forming Ir‐O‐C, which leads to high stability and enhanced activity for acidic HER/OER.^[^ ^36^ ^]^ 2) Unsaturated coordination sites are suggested to be created on substrates, such as graphitized carbons and Ti‐/W‐based compounds. These unsaturated coordination sites, e.g., vacancy and dangling bond, have strong interactions with active metals, thus resulting in better stability.

## 5 Design of Fluid Homogeneous Electrolysis System

Beyond designing atomic/nano structures of heterogeneous electrocatalysts and corresponding electrode systems, as discussed above, homogeneous electrocatalysis systems can be used for water electrolysis. Corresponding homogeneous electrocatalysts with properties that can be tuned controllably have been developed with inspiration from natural enzymes. The hydrogenases, consisting of iron‐ and/or nickel‐based compounds, can catalytically produce H_2_ from water at their metal active centers.^[^ ^147^ ^]^ A typical example of O_2_ evolution in nature is found in photosystem II, where the multinuclear Mn_4_O_4_ clusters of CaMn_4_O_5_ are the active center for water oxidation.^[^ ^148^ ^]^ The Mn_4_Ca core is another active center for oxygen evolution.^[^ ^149^ ^]^ To mimic these active centers with enhancement, various types of homogeneous catalysts have been designed, most of which are based on organometallic complexes with different metal‐organic ligand coordinations and ligand structures. Moreover, some inorganic soluble homogeneous electrocatalysts, such as polyoxometalate^[^ ^150^ ^]^ and polycarbonyl compounds^[^ ^151^ ^]^ have been developed. Importantly, homogeneous catalysts are soluble in specific solutions (normally mixed solvent), thus enabling the design of new fluid homogeneous electrocatalytic systems in which the soluble electrocatalysts on the anode and cathode can be resupplied and recycled after performance attenuation (see Figure 8); this system favors SHP. Here, we review the significant advances in homogeneous catalysts in terms of HER and OER and highlight the current issues as well as the strategies used to fabricate efficient fluid homogeneous electrolysis systems.

**Figure 7:**
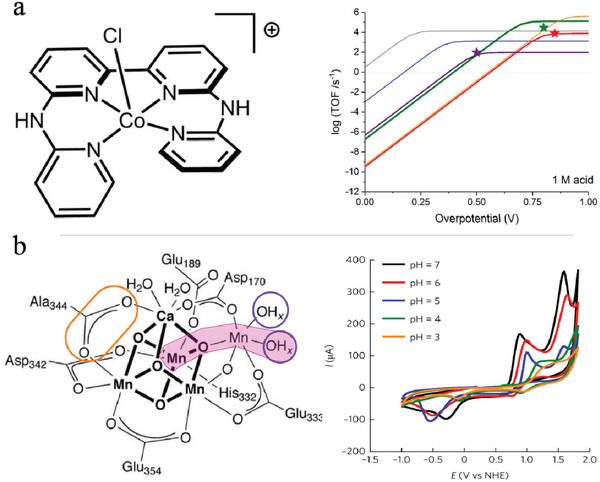
a) Structure of [Co(bapbpy)Cl]^+^ and catalytic Tafel plots in relation to turnover frequency (s^−1^) for HER in 1 m HBF_4_ (bold red and purple traces) and 1 m HNEt^3+^ (bold green trace), in comparison with other molecular catalysts: [Ni(P_2_PhN_2_C_6_H_4_CH_2_P(O)(OEt)_2_)_2_]^2+^ (blue trace), [Fe(TPP)Cl] (orange trace) and [Co(dmgH)_2_(py)] (grey trace). Adapted with permission.^[^ ^156^ ^]^ Copyright 2020, American Chemical Society. b) Structure of [Mn_12_O_12_(O_2_CC_6_H_3_(OH)_2_)_16_(H_2_O)_4_] and cyclic voltammograms in 0.1 m acetate buffer at different pH values. Adapted with permission.^[^ ^164^ ^]^ Copyright 2017, Nature Publishing Group.

Among the various metal‐based homogeneous HER electrocatalysts, the Co‐, Ni‐, and Fe‐based materials^[^ ^152^, ^153^, ^154^, ^155^ ^]^ are extensively studied because they have high intrinsic activity. The electrodes of glassy carbon (GC), mercury, graphite, and gold amalgam are normally used to transfer electrons from the electrode to the catalyst. Either mercury or gold amalgam, with high HER overpotentials, are suitable for a more accurate evaluation of the HER activity of homogeneous electrocatalysts;^[^ ^152^ ^]^ the GC electrode is also widely applied because its excellent chemical stability. Artero et al. developed a homogeneous [Co(bapbpy)Cl]^+^ (bapbpy refers to 6,6’‐bis‐(2‐aminopyridyl)‐2,2’‐bipyridine) electrocatalyst (**Figure** **7**a) on a GC working electrode for H_2_ production from aqueous solution, where the potential and proton strength influence the HER reactivity/stability.^[^ ^156^ ^]^ The performance of the catalyst during HER was examined in the presence of HBF_4_ or Et_3_NHBF_4_ as the proton source, wherein TOF reached 110 s^–1^ at the ion concentration of 1 m H^+^, as shown in Figure 7a. By evaluating stability using transfer change and time as parameters, strong acid was found to increase the catalyst lifetime, and the applied potentials were found to correlate with catalyst stability because they determine the formation of active intermediates. Typically, highly reduced active intermediates generated at high overpotentials, e.g., CoII hydrides and ligand‐reduced species, may reduce catalyst stability. Another Co‐based molecule complex, [Co(Fc‐tpy)_2_](PF_6_)_2_, was found to electro‐catalyze the evolution of H_2_ in a DMF/H_2_O solution (95:5 v/v) containing acetic acid as the proton source, yielding a high TOF of 825 s^–1^.^[^ ^157^ ^]^ The cobalt complex consists of a pentadentate ligand and can be used at a solvent mixture containing 50% water, delivering a rate constant of 70 s^−1^ for HER.^[^ ^158^ ^]^ In addition to Co‐based complexes, molecular complexes produced by combining nickel with bis(diphenylphosphanyl)amine ligands were demonstrated to be potential homogeneous catalyst for HER, with high TOF (2827–5149 s^−1^) and stability in acidic conditions.^[^ ^159^ ^]^ Ni molecular complexes consisting of bis(diphenylphosphanyl)amine ligands also have excellent stability but with relatively lower TOF 368.59–585.17 s^–1^ in trifluoroacetic acid‐containing solutions.^[^ ^160^ ^]^ Moreover, Cu‐based^[^ ^161^, ^162^ ^]^ and metal‐free molecular complexes, e.g., 2,4,6‐triphenylpyridinium perchlorates,^[^ ^163^ ^]^ have been developed for HER. In general, although high TOF values are obtained with these homogeneous molecule catalysts, the overall activity is still significantly lower than in the presence of heterogeneous catalysts, as discussed above. Moreover, the conditions used to test the stability of homogeneous catalysts are less severe than in the case of heterogeneous catalysts. For example, the pH values for most tests on homogeneous molecule catalysts are higher than 2 because stronger acids may induce their decomposition or corrosion.

As alternative metals, Ru, Ir, Co, Mn, Fe, Ni, and Cu are highly active species for homogeneous OER.^[^ ^39^, ^148^, ^165^, ^166^ ^]^ However, many of these competent molecular catalysts,^[^ ^167^, ^168^, ^169^, ^170^, ^171^ ^]^ e.g., the most typical homogeneous Cu complexes,^[^ ^165^ ^]^ are usually tested under neutral or alkaline conditions, making them inappropriate for SHP in acidic media. In 2017, a soluble Mn cluster was reported by Maayan et al. for homogeneous OER.^[^ ^164^ ^]^ The Mn complex, defined as [Mn_12_O_12_(O_2_CC_6_H_3_(OH)_2_)_16_(H_2_O)_4_] (see Figure 7b), can be stabilized in aqueous weak acid (acetate buffer pH 6), yielding an overpotential of 334 mV for OER on the GC electrode. The hydroxyl substituents on the ligands contribute to catalysis activity and also the solubility and stability of the complex in aqueous solutions. The authors further improved this manganese cluster and synthesized a [Mn_12_O_12_(O_2_CC_6_H_2_(OH)_3_)_16_(H_2_O)_4_] complex,^[^ ^172^ ^]^ which facilitated water oxidation with an ultra‐low overpotential of only 74 mV, turnover number (TON) of 13.2, and high Faraday efficiency (FE) of 93%. Although these two Mn‐based complexes can be used in aqueous solutions, most transition metal complexes require an organic/water mixed solvent for their stability and solubility. Another prime example of homogeneous electrocatalysts for OER is the polyoxometalate‐based compounds (Mo‐, W‐, V‐based polyoxometalates).^[^ ^173^, ^174^ ^]^ Compared with the organic ligands in molecular catalysts for OER and HER, the ligands in polyoxometalates are inorganic and hard to oxidize during OER, making the polyoxometalate‐based compounds stable and promising for homogeneous OER applications.^[^ ^174^ ^]^ The high‐valent metals in the pure polyoxometalates are usually not sufficiently active, which has driven abundant studies on the incorporation of polyoxometalates with various transition metals to tune their redox properties.^[^ ^175^ ^]^ However, most current studies on metal‐incorporated polyoxometalate compounds are conducted under photochemical and/or nonacidic conditions.^[^ ^173^ ^]^ This is probably because the instability and/or non‐activity of these compounds under acidic conditions^[^ ^175^, ^176^ ^]^ make them unsuitable for SHP in acidic media.

For highly stable and active soluble molecular catalysts, the fundamental design targets are the coordination and geometric structures and the transition metal species:^[^ ^148^ ^]^ Active metals with coordination sites and labile chemical states are usually selected because they have high intrinsic reactivity. Moreover, highly stable ligand scaffolds are preferable because they can stabilize metal active sites with different oxidation/reduction states. In particular, ligands containing heteroatoms, such as N, S, and P are electron withdrawing/donating, and their redox properties promote electrocatalytic activity.^[^ ^148^ ^]^ Despite the principles and advances discussed above, some notable issues still exist that impede the application of homogeneous catalysts for SHP in acidic media: 1) First, the stability of most homogeneous electrocatalysts for both HER and OER is poor. Most reported electrocatalysts cannot endure the aqueous solution environment, especially the strongly acidic conditions (pH < 1), because even the robust homogeneous catalysts highlighted above decompose under these conditions. In this regard, some acid‐stable molecular catalysts used in photosynthesis, such as [Ru(bda)(isoq)_2_]^[^ ^177^ ^]^ and [Fe(CF_3_SO_3_^–^)_2_(^Me2^Pytacn)],^[^ ^178^ ^]^ are worth developing for electrochemical systems. 2) The efficiency of known homogeneous molecular catalysts working in acidic electrolytes is rather low, as illustrated by the large overpotentials and low Faradaic yields (or FE).^[^ ^148^, ^152^, ^179^ ^]^ The current densities obtained during HER/OER are generally several orders of magnitude lower than those with heterogeneous electrocatalysts. Normally, organic solvents (e.g., CH_2_Cl_2_, DMF, THF, CH_3_CN, and DMSO) are used to ensure the solubility and possibly the stability of homogeneous catalysts,^[^ ^152^ ^]^ although they lack the proton source for HER and OER, thus limiting efficiency. Moreover, the conductivity of most homogeneous catalysts for water splitting is very poor.^[^ ^148^ ^]^ Both these factors limit efficiency. 3) The remaining challenges include the separation of active homogeneous catalysts from the decomposed ones after use and the regeneration of homogeneous catalysts.

**Figure 8:**
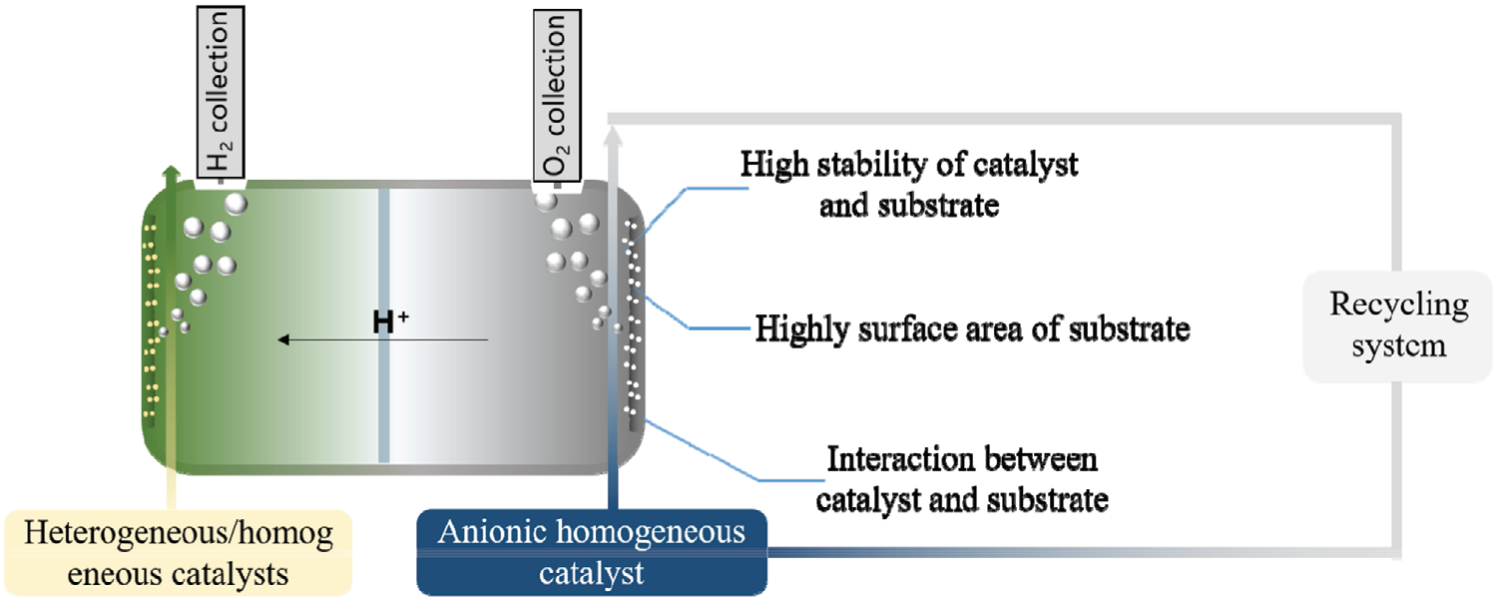
Schematic diagram of the fabrication of efficient fluid homogeneous electrolysis systems for SHP.

Here, we propose a concept for fluid homogeneous electrolysis systems to partially address these issues toward SHP, as illustrated in **Figure** **8**. In addition to using highly stable and active homogeneous catalysts, as aforesaid, several factors can be further considered to improve this system: 1) The electrode substrate provide active sites for homogeneous reaction. Compared with the HER side, the electrode substrates of the anode have higher stability requirements to withstand the more severe OER processes. For this purpose, metal oxides or highly graphitized carbon can be considered as substrates to mitigate oxidative degradation.^[^ ^175^ ^]^ 2) Porous structures with a large surface area and high conductivity are recommended for electrode substrates. Homogeneous reactions are fast in the presence of electrode substrates that can effectively transfer electrons to/from molecular catalysts, and porous structures with a large surface area can ensure fast mass transfer and the concurrence of more reactions to maximize activity. 3) Heterogeneity can be achieved by immobilizing molecules on electrode substrates with/without other solid supports; this can address issues related to stability, activity, and also separation. Immobilization can be achieved via covalent or noncovalent interactions with improved charge transfer.^[^ ^148^ ^]^ For example, immobilization using noncovalent interactions can be achieved via weak interactions, such as CH−*π* interactions, as demonstrated by the Ru oligomer and CNT.^[^ ^180^ ^]^ Other graphitic‐type carbons can also be used as electrode substrates for immobilization. To enable stronger immobilization of molecular catalysts, covalent linkages are usually needed, which requires delicate surface functionalization of electrode substrates/supports to provide the groups or defects (e.g., COOH, SH, OH, PO_3_H_2_, NH_2_, and pyrene)^[^ ^147^, ^181^, ^182^, ^183^ ^]^ for anchoring. Typically, [Ru(C_8_Otpy)(H_2_dcbpy)(OH_2_)]^2+^ is anchored on the nanoporous TiO_2_ surface via the interaction between carboxylic groups and surface oxides.^[^ ^181^ ^]^ This complex is stable during OER with a high current of 0.39 mA cm^–2^ and FE of 83%. In general, immobilization should be strong enough to retard the activity loss of molecule during electrocatalysis, and it should not alter the physical and chemical nature of molecular catalysts or isolate them, which limits their original electrocatalytic function. 4) A recycling system, capable of replenishing homogeneous catalysts after partial degradation and also of separating active and decomposed catalysts, is worth further exploration. In designing advanced molecule catalysts for this system, operando characterizations are requisite to analyze the molecular structure and the catalyst‐substrate/support interactions. Catalysts should exhibit a large difference in interactions before and after electrocatalysis to facilitate the separation of active and decomposed components.

## 6 Decoupling/Hybridizing‐Governed Sustainability

**Figure 9:**
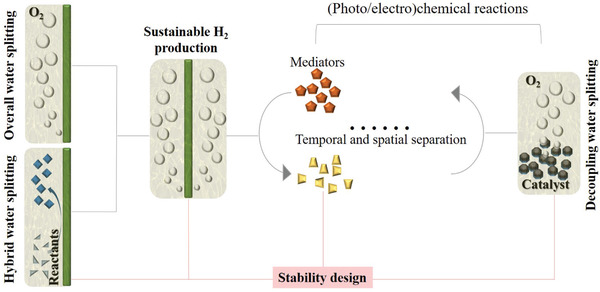
Schematic diagram of the fabrication of overall, hybrid and decoupling water electrolysis systems for SHP.

In addition to designing more stable materials/electrodes and optimizing devices, controlling reaction type is another important strategy to promote the stability of catalysts/electrodes toward SHP. For example, by replacing anode OER with (ir)organic‐molecule oxidation reactions requiring lower potentials than OER (organic‐molecules such as 5‐hydroxymethylfurfural and benzyl alcohol),^[^ ^184^, ^185^ ^]^ the electrocatalyst should be more stable and durable under the corresponding mild electrochemical conditions. Therefore, new hydrogen generation systems should be designed with the aim of replacing anode reactions with new reactions (hybrid strategy) or coupling new (electro)‐chemical reactions with original reactions (decoupling strategy) (see **Figure** **9**). Here, in a decoupling strategy, hydrogen evolution is not directly coupled with oxygen evolution. It needs redox mediators (solid or soluble states), which serve as reversible electron/proton acceptors/donors and can pair with hydrogen evolution to form a new coupling reaction. In this way, HER and OER are temporally and spatially separated. Thus, the decoupling strategy endows hydrogen evolution with some distinguishing features, i.e., faster hydrogen production rate by employing a fast pairing reaction; higher hydrogen purity and safety by eliminating O_2_ mixing; better operation flexibility based on the separation of time and space; and benefits of coupling unstable renewable energy sources.^[^ ^12^, ^20^ ^]^ More importantly, the decoupling strategy disassembles the HER process from the rigorous OER process, thus enabling the design and optimization of (electro)‐catalysts/electrodes independently toward higher stability (see Figure 9). Hybrid water electrolysis can be deemed as the “half” of decoupling water electrolysis, and thus it has some similarity with the decoupling process, except the pairing reaction usually refers to irreversible but thermodynamically favorable oxidation of (ir)organic molecules (see Figure 9). Typically, electro‐oxidation of alcohol, amine, aldehyde, hydrazine, and urea was widely used to replace the OER process at lower potential conditions in the corresponding hybrid water electrolysis systems.^[^ ^186^ ^]^

In a decoupling system, two primary factors influence operation stability and thus SHP: 1) the first influencing factor is the stability of mediators (see Figure 9). There are many kinds of mediators that can be used in the decoupling system, including polyoxometalate decoupling agents (e.g., phosphomolybdic acid and silicotungstic acid),^[^ ^187^, ^188^ ^]^ transition metal redox mediators (e.g., vanadium salts and iron complexes),^[^ ^189^, ^190^ ^]^ soluble organic redox mediators (e.g., quinone‐based materials),^[^ ^191^ ^]^ and solid state redox mediators (e.g., NiOOH/Ni(OH)_2_ electrodes and polytriphenylamine).^[^ ^192^, ^193^ ^]^ Among these candidates, solid‐state inorganic redox mediators dissolve in strong acids and cannot be used directly under these conditions. Ideal mediators should meet the requirements of highly stable reduced and oxidized forms, highly reversible redox cycles to achieve high Faraday and energy efficiencies, and limited possible degradation or side reactions, both of which are unfavorable for SHP. The poor stability of mediators, especially soluble redox mediators, may be partially resolved with a replenishment strategy that is analogous to the continuous flow system aforementioned. 2) The second influencing factor is the stability of (electro)‐catalysts/electrodes in the decoupling system, which is largely determined by the redox mediators and reaction type (Figure 9): electrochemical−electrochemical, electrochemical−chemical, or electrochemical−photo(electro)chemical reactions.^[^ ^20^ ^]^ In a decoupling system using highly reversible redox mediators, the redox reaction rate of the mediators can be tuned by increasing the dosage to levels higher than that of HER/OER. Thus, activity limitations can be overcome, and stability can be optimized by choosing highly stable and moderately active materials. For example, for the electrochemical−chemical decoupling system using silicotungstic acid as the mediator, all the MoS_2_, Mo_2_C, Ni_5_P_4_, and Ni_2_P catalysts show activity for hydrogen production.^[^ ^194^ ^]^ Therefore, it is possible to select the one with the highest stability. In a hybrid system, the system configuration was normally simpler as compared to most decoupling systems, and the factors to be considered in association with stability were fewer. There is no need to consider the stability of (ir)organic reactants. However, the (ir)organic reactants‐related reaction types, the corresponding electrode catalysts, and the system configuration are regarded as the main conditions to determine the operation stability of a hybrid system toward SHP.

**Figure 10:**
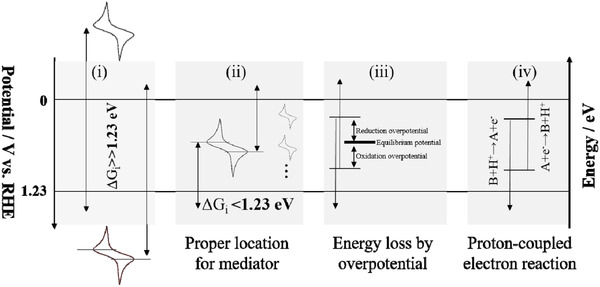
Design principles determined from energy scenarios of different redox mediators and the factors influencing decoupled water splitting relative to conventional water splitting.

Studies on decoupling/hybrid systems are rarely focused on stability, and direct comparison of detailed examples cannot be made to obtain the optimal mediators, reactants, (electro)‐catalysts/electrodes, and/or system types. Here, we present the current situation and several design principles for the decoupling/hybrid systems of SHP (see **Figure** **10**): 1) From the viewpoint of overall energy, it is disadvantageous to select a mediator with an oxidation potential far below 0 V versus RHE and with a reduction potential far above 1.23 V versus RHE in a decoupling system. This is because Δ*G* is significantly larger than 1.23 eV in these cases, leading to a high voltage for the electrochemical process. Similarly, the electro‐oxidation of (ir)organic reactants with the electrode potentials close to or even higher than OER potentials should be avoided in a hybrid system. In both cases, the liability of mediators and/or (electro)‐catalysts/electrodes is unfavorable for SHP. 2) In a decoupling system, one of the roles of mediators is to resolve Δ*G* into several Δ*G*_i_ for overall water splitting, as long as their sum is equal to Δ*G*, which is larger than 1.23 eV.^[^ ^20^ ^]^ The potentials for the reversible redox coupling of a mediator should be located between the two onset potentials of HER and OER to resolve the Δ*G*_i_ of each electrolytic step to values below 1.23 eV. Using multiple mediators, Δ*G*_i_ can be further reduced. In this way, the redox reaction of each electrolytic step should be mild and stable because of the highly reversible mediators and fast redox processes, facilitating SHP. 3) The redox processes of mediators/reactants usually require reaction energy barriers, represented by the additional overpotentials or other energy input, such as thermal and photo energy,^[^ ^20^ ^]^ thus increasing the energetic cost. Both the use of highly reversible mediators or highly reactive reactants, and the introduction of efficient catalysts in the decoupling/hybrid systems can reduce superfluous energy consumption and make the decoupling/hybrid processes more effective for SHP. 4) Changes in pH may be a severe constraint on stability/durability of (electro)‐catalysts/electrodes in decoupling systems.^[^ ^12^ ^]^ Therefore, it is better to use mediators with pH buffering effects, which can usually accept protons during OER and release protons during HER. Thus, pH fluctuations can be prevented during decoupling processes, which is also crucial for SHP in practical electrolyzers. 5) Current electrocatalysts used in membrane‐free hybrid water electrolysis systems would be a challenge for acidic SHP. This is because most of the advanced materials, such as NC‐coated Ni‐Mo‐N nanowire,^[^ ^195^ ^]^ and Ni, Co hydroxide,^[^ ^196^ ^]^ were only used in alkaline media and probably not acid‐stable. Therefore, there leaves a great room for exploiting the acid‐stable electrocatalysts, especially the noble metal‐based catalysts for the acidic hybrid systems. (6) Application of suitable membranes can allow the resolution of stability issues of anode materials for decoupling/hybrid systems, by individual optimize the anolyte and counterpart reactions of acidic HER. In this case, however, the challenge is changed to the membrane stability, and highly stable membrane should be applied.

## 7 Challenges and Outlook

We have reviewed the latest advances in water electrolysis, summarized and proposed four comprehensive strategies for SHP based on water electrolysis, and resolved the related features, merits/demerits, and current situation of each strategy. Most of the strategies are still in their infancy, yet many opportunities are worth further investigation. The challenges of these strategies and the corresponding opportunities for future SHP applications are described below.

Designing new catalysts for SHP in acidic media is a general strategy that is highly reliant on the stability and activity of electrode materials. The stability and activity of most non‐noble metal materials are inferior to those of noble metal ones, especially for acidic OER. Moreover, even anode noble metals find it hard to satisfy the requirements of practical acidic water electrolysis (2–3 A cm^–2^ for a minimum of 50 000 h).^[^ ^17^ ^]^ The high current and long operation time in commercial applications not only require the catalytic electrode to have high mechanical stability with excellent resistance to gas impact, but also intrinsic stability to endure the corrosion dissolution. We have exemplified various effective pathways to enhance the intrinsic stability and activity of electrode catalysts; these pathways include surface modification/protection, strain modulation, phase engineering, support interaction, morphology and facet controls, and heteroatom doping. In particular, tuning multi‐metal centers can be effective at promoting activity, and controlling the multidimensional structure by jointly optimizing the atomic and electronic structures may further enhance the stability of electrocatalysts. Among the electrocatalysts for acidified water electrolysis, the Ru‐, Pd‐, Mo‐, and Ni‐based materials show great potential for acidic HER, whereas the Ir‐ and Ru‐based materials are still the preferred candidates for acidic OER. Therefore, multidimensional structure engineering can be conducted on these multi‐metal complex to achieve ideal acidified water electrolysis performance. For example, by phase engineering the Ir‐M (M = Fe, Co, Ni, Mn, etc.) complex oxides of perovskites, high stability may be achieved because the coordination of metal sites is strong in a highly crystalline phase, thus retarding metal dissolution in acidic media. Moreover, high activity via optimized electron modulation can lead to accelerated intermediate reactions.

Fabricating symmetrical electrode systems is a strategy that is guided by individual electrode catalyst design, which necessitates the use of bi‐functional electrocatalysts, such as Ru‐Ni, Ir‐Co, and other multicomponent alloys. For component selection, metal ions (e.g., Co and Fe) that can induce the formation of radical oxygen species from peroxide to decompose polymer backbones and degrade membranes^[^ ^14^ ^]^ should be reevaluated carefully because they may also reduce the stability of the system against SHP. Additionally, strong interactions between active bi‐functional metal components and robust electrode substrates (e.g., graphitized carbon and Ti‐/W‐based electrodes) are crucial for the stable operation of this system at a low exchange frequency. Such strong interactions are also of profound importance in fluid homogeneous electrolysis systems, which can provide a heterogenization route to stabilizing homogeneous molecular catalysts and guaranteeing their accessibility. In particular, electrode substrates/supports with high conductivity, porous structures, and large surface areas facilitate accelerated electron transfer and mass transportation, and they also support a high number of adsorbed molecular catalysts to maximize water electrolysis activity. In addition, the heterogenization route is a basis for the recycling of molecular catalysts via the fine design of differentiated adsorption between substrates and active/decomposed molecules. Even with the aid of heterogenization, the structural evolution of molecular catalysts can still occur in electrocatalytic processes, and most catalytic activities are still insufficient to meet the requirements of commercial water electrolysis. Therefore, more effective heterogenization methods should be developed. For example, the functionalization of substrates/supports to impart synergistic HER/OER activities or the construction of optimized bond modes to facilitate electron transfer (e.g., ‐M‐N‐N‐M bridging mode)^[^ ^174^ ^]^ may enhance activity/stability for water electrolysis toward SHP.

Beyond the structural evolution of molecular catalysts, heterogeneous electrocatalysts, even the robust ones, are not absolutely unsusceptible to phenomena that occur during their applications, i.e., agglomeration of single atom catalysts and cation leaching combined with the reconstruction of Ir‐based perovskites. Consequently, accurate determination of catalyst structure, active centers, and the underlying mechanism of electrolysis is essential for uncovering the guiding principles conductive to actual electrode catalyst design. In considering the dynamic processes of interconvertible anode and cathode systems, fluid homogeneous catalytic systems, and some decoupling/hybrid systems, in situ/operando characterization techniques should be developed to monitor the dynamically structural and component changes. Among these techniques, in situ/operando electron microscopy, X‐ray absorption spectroscopy, and X‐Ray diffraction techniques are powerful tools to survey these dynamic processes. In particular, a combination of in situ/operando X‐ray spectroscopy and electron microscopy can facilitate the identification of dynamic active sites as well as the surface reaction processes of interconvertible anode and cathode systems or fluid homogeneous catalytic systems. With these dynamic surface‐related information, rational design of catalysts and systems can be achieved for SHP.

**Figure 11:**
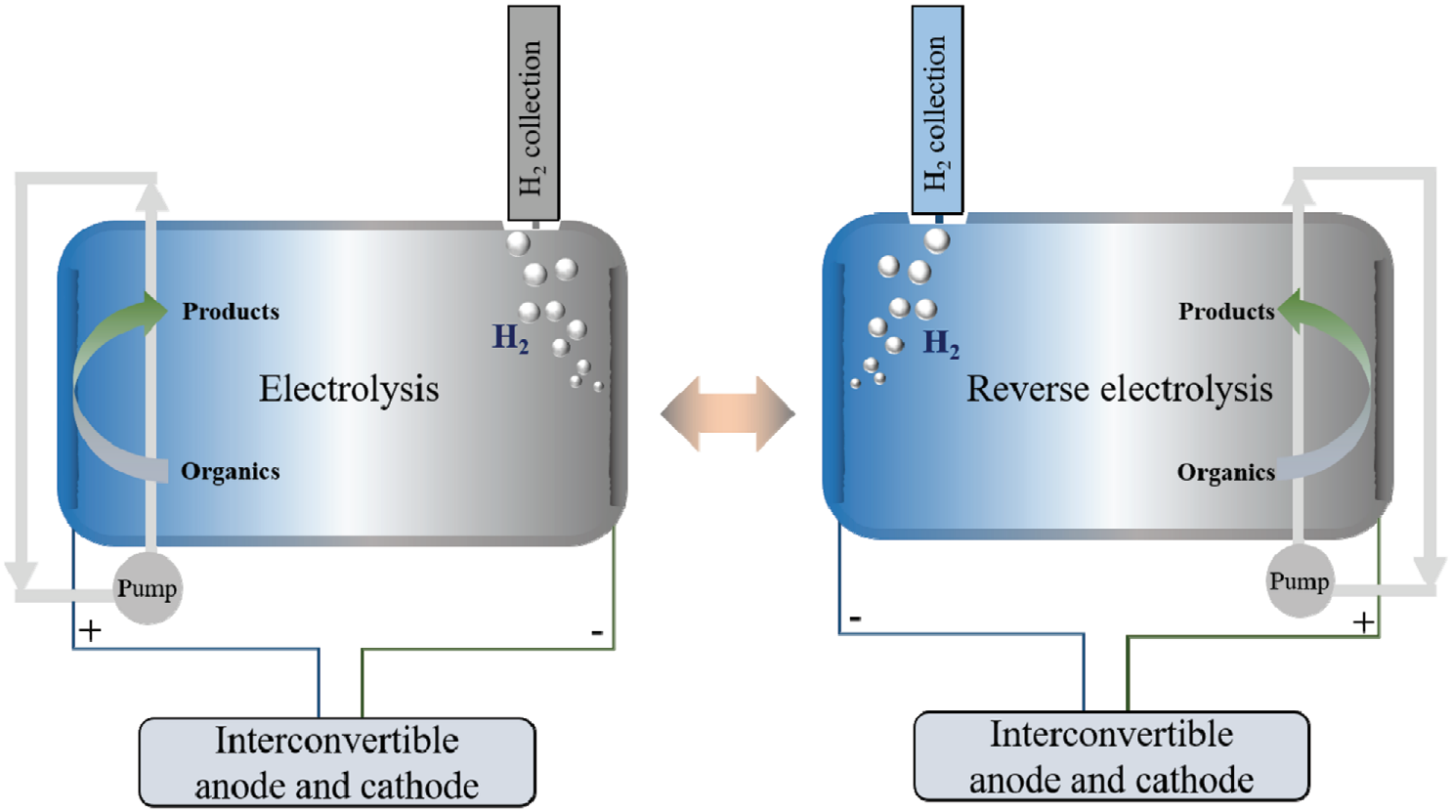
Schematic diagram of the fabrication of membrane‐free hybrid water electrolysis systems with interconvertible electrodes for SHP.

The thermodynamics reactions of decoupling and hybridizing water electrolysis controls are more favorable for SHP. To develop such systems with higher sustainability for H_2_ production, two factors should be considered: First, the intrinsic properties of catalysts and reactants should be enhanced. For example, high stability, solubility, and fast electron transfer ability are desirable for redox mediators in decoupling systems and high selectivity for the electrooxidation of organic/inorganic reactants (e.g., various biomass‐derived intermediates, H_2_S, SO_2_, Cl^–^) to desired products is required of hybridizing systems. These aspects can enhance the overall performance of water electrolysis. Second, the composite cost of these two systems should be reduced, including the synthesis cost of mediators, cost of system construction (i.e., catalysts, proton exchange membrane), and cost of operation. Highly active and stable electrodes/catalysts should be used to reduce the overpotential, and device configuration, reactions steps, and post‐processing processes (e.g., separation and purification) should be simplified to increase energy efficiency and reduce operation cost for SHP. A promising system for SHP can be achieved by further integrating the advantages of different strategies. For example, a new hybrid system with interconvertible electrodes is proposed as shown in **Figure** **11**. The electrode electrocatalyst should be highly stable and active during HER, and it should be selective for and active during organic oxidation but inactive during the reduction of oxidized products. With the aid of flow pumps and periodic regeneration of anodes/cathodes, long‐term SHP can be achieved in this hybrid membrane‐free system.

In summary, the development status, features, challenges, and opportunities of the four strategies for SHP were summarized and outlined in this review. It is hoped that this review will stimulate comprehensive development of these strategies and that technological difficulties will be overcome by transcending the prevailing paradigm of water electrolysis for SHP. With continued research and development in this field, acidified water electrolysis would be demonstrated as an influential technique for SHP toward solving environmental and energy issues.

## Conflict of Interest

The authors declare no conflict of interest.
